# Combined Liuzijue and mindful breathing promote greater sustained relaxation than mindful breathing alone in university students with anxiety symptoms

**DOI:** 10.3389/fpsyg.2026.1854234

**Published:** 2026-07-13

**Authors:** Wen-Ming Liang, Inga Griškova-Bulanova, Jing Xiao, Qi Zhao, Ming-Ying Hou, Fei-Fei Ren, Yang Zhu, Inga Truskauskaitė, Yu-Han Xiao, Osvaldas Rukšėnas

**Affiliations:** 1 Physical Education Institute, Jimei University, Xiamen, China; 2 Life Science Center, Vilnius University, Vilnius, Lithuania; 3 Xiyuan Hospital, China Academy of Chinese Medical Sciences, Beijing, China; 4 Department of Physical Education, Beijing Language and Culture University, Beijing, China; 5 School of Sports Medicine and Rehabilitation, Beijing Sport University, Beijing, China; 6 Institute of Psychology, Vilnius University, Vilnius, Lithuania; 7 School of Traditional Chinese Medicine, Shanghai University of Traditional Chinese Medicine, Shanghai, China

**Keywords:** anxiety, heart rate, heart rate variability, Liuzijue breathing, mindful breathing, relaxation

## Abstract

**Background:**

Mindful breathing is a well-established method for inducing relaxation, but anxious individuals often struggle to enter a mindful state. Liuzijue breathing, a traditional Chinese practice involving controlled abdominal movements, low-pitched vocalization, and guided mental focus, may facilitate mindful breathing and enhance its efficacy. This study examined whether combining Liuzijue breathing with mindful breathing could help anxious students achieve relaxation more effectively.

**Methods:**

Twenty-four male participants (aged 21 ± 2, Self-Rating Anxiety Scale score 57 ± 8) completed this randomized crossover trial with two experimental sequences on consecutive days: Sequence A: pretest, 15 min mindful breathing, posttest; Sequence B: pretest, 5 min Liuzijue breathing followed by 10 min mindful breathing, posttest. Heart rate and heart rate variability were recorded as indicators of relaxation, and subjective concentration levels during mindful breathing were assessed. In an additional session, respiration rate (RR), peripheral oxygen saturation (SpO₂), and end-tidal carbon dioxide (EtCO₂) were measured during 5 min of breathing practice and a 3 min rest period.

**Results:**

Compared with 15 min of mindful breathing alone, the combination of 5 min Liuzijue breathing followed by 10 min mindful breathing produced more sustained relaxation. Significant interaction effects were observed for heart rate, which showed a greater continued reduction, and for heart rate variability in both time and frequency domains, which showed greater progressive increases. Concentration ratings during mindful breathing were also significantly higher following Liuzijue breathing. RR was lower during Liuzijue breathing and remained significantly reduced during the first 2 min of rest. SpO₂ was significantly higher during Liuzijue breathing and at the first minute of rest. EtCO₂ was significantly lower from minutes 2–5 of Liuzijue breathing, with no differences observed during the 3 min resting period.

**Conclusion:**

The combination of Liuzijue and mindful breathing produced longer-lasting relaxation than mindful breathing alone in male university students with anxiety symptoms. The reduced RR and elevated SpO₂ induced by Liuzijue breathing may partly explain this effect. These findings suggest that Liuzijue breathing may serve as an effective preparatory technique to optimize mindfulness-based interventions.

## 1 Introduction

Mental health problems are highly prevalent across the world among university students (Ketchen Lipson et al., 2015; Beiter et al., 2015; Fernández-Rodríguez et al., 2019), with anxiety being among the most common (Pedrelli et al., 2015). In China, high rates of anxiety have been reported. A survey covering 15 universities in China and involving 1,892 undergraduate students found that anxiety was not only widespread but also a problem of considerable severity (Gao et al., 2020). Another survey of 1,017 first-year Chinese university students revealed a prevalence rate of anxiety (ranging from mild to severe) of 40.3% (Gao et al., 2021). More recently, nearly half of Chinese college students were found to have anxiety tendencies or symptoms (Han et al., 2025). Anxiety has been associated with distress, poor academic performance, and reduced job prospects after graduation (Chu et al., 2023; Moghimi et al., 2023). These findings highlight the urgent need to prevent and alleviate anxiety in this population.

Enhancing relaxation and shifting the autonomic nervous system toward a ‘rest-and-digest’ state are effective approaches to reducing anxiety (Kim and Kim, 2018; Magnon et al., 2021), while mindfulness practice is widely used to promote relaxation and enhance parasympathetic activity (Li et al., 2023; Brown et al., 2021). A meta-analytic review of 39 studies with over 1,100 participants showed that mindfulness-based therapy has a moderate to large effect size in reducing anxiety symptoms, and the effects were maintained over follow-up (Hofmann et al., 2010). A frequently referred definition of mindfulness is “paying attention in a particular way: on purpose, in the present moment, and non-judgmentally.” (Kabat-Zinn, 2005). Mindfulness practice is often conceptualized through three core components: intention, attention, and attitude. Intention refers to the underlying purpose or motivation for practicing mindfulness, which directs the focus of practice and aims to cultivate greater awareness (Batchelor, 2011). Attention involves observing your internal and external experiences as they occur in the present moment (Shapiro et al., 2006). Attitude is characterized by a non-judgmental approach (Hall-renn, 2007). There are various methods to practice mindfulness, with mindful breathing being a popular one. Mindful breathing requires practitioners to be aware of the breath, without controlling its speed and depth, and without judging its quality (Burg and Michalak, 2011). One study found that both mindful breathing practice and cognitive reappraisal practice yielded large effect sizes in reducing test anxiety, with mindful breathing scoring significantly higher on positive thoughts than both cognitive reappraisal and control conditions (Cho et al., 2016). Another study revealed that even 5 min mindful breathing could significantly reduce distress (Beng et al., 2016).

However, individuals with anxiety may experience difficulty entering a mindful state. Based on our teaching observations, students exhibiting anxiety-related behaviors (e.g., nervousness, restlessness) frequently report challenges in maintaining attention during the mindful breathing exercise. Research has shown that, compared to individuals without anxiety disorders, those with generalized anxiety disorder tend to experience greater difficulties in emotion regulation and exhibit lower levels of mindfulness (Moghimi et al., 2023). Worry is a core feature of anxiety; a study has found that individuals who tend to worry may experience difficulty engaging in mindfulness practice (Banerjee et al., 2018). The above evidence suggests that individuals with anxiety may initially find mindfulness practice more challenging. Therefore, it is necessary to explore methods that can help people with anxiety enter a mindful state more easily and quickly, in order to enhance the effectiveness of mindfulness practice.

Six Healing Sounds Qigong (Liuzijue) has the potential to effectively enhance the practice of mindful breathing. Qigong is a traditional Chinese practice that integrates physical movement, breath regulation, and mental focus to optimize energy (Qi) (Klein et al., 2017). Liuzijue is recognized as one of the most popular forms of Qigong in China, and it is unique among Qigong systems in that it primarily features vocalized breathing exercises (Tang et al., 2021; Zhang et al., 2022). There are six sounds in Liuzijue practice: xu, he, hu, si, chui, and xi. The detailed procedure of Liuzijue breathing is described below, using “chui” as an example. During inhalation, the rib cage expands laterally, the upper abdomen (between the navel and the xiphoid process) relaxes, and the lower abdomen (from the navel to the pubic symphysis) contracts. During exhalation, the inspiratory muscles gradually relax and a low-pitched sound is produced. During vocalization, attention should be directed from the mouth and throat to the chest, and subsequently to the lower abdomen (Health Qigong Administrative Center of the General Administration of Sport of China, 2021). Liuzijue breathing may help individuals quickly enter a calm and focused state for the following reasons: (1) the Liuzijue breathing technique requires the brain to consciously control the contraction and relaxation of the respiratory muscles, which helps anchor mental activity to a specific focus; (2) the low-pitched sound produced during exhalation promotes mental relaxation (Simpson et al., 2021); (3) the breathing pattern used in Liuzijue is characterized by slow, controlled breathing, which has been shown to enhance parasympathetic activity (Radaelli et al., 2004; Laborde et al., 2022); (4) in Liuzijue, the exhalation phase is slower and longer than the inhalation phase, which may further stimulate parasympathetic activation (Bae et al., 2021; Laborde et al., 2021; Strauss-Blasche et al., 2000). Nevertheless, Liuzijue breathing practice is typically limited to 5–10 min, as it requires intentional mental engagement and the coordinated activity of respiratory muscles and vocal organs, which can lead to fatigue with extended practice. In contrast, mindfulness breathing can usually be sustained for a longer duration (Lim et al., 2024).

Thus, combining Liuzijue with mindful breathing may enhance relaxation among anxious students. Heart rate (HR) and heart rate variability (HRV) serve as direct indicators of relaxation and autonomic nervous system activity. Regarding HRV variables, the time-domain measure, Root Mean Square of Successive Differences (RMSSD, ms), primarily reflects short-term parasympathetic (vagal) activity, while High-Frequency power (HF, ms^2^; 0.15–0.40 Hz) is a frequency-domain measure of parasympathetic activity (Laborde et al., 2017). Both RMSSD and HF are recommended as core HRV indices (Laborde et al., 2017), with higher values indicating stronger parasympathetic activity. This study, therefore, aimed to investigate whether 5 min Liuzijue breathing followed by 10 min mindful breathing would yield lower HR and higher HRV, as reflected by RMSSD and HF, relative to 15 min mindful breathing alone.

## 2 Methods

The study was conducted in accordance with the Declaration of Helsinki and approved by the Ethics Committee of Jimei University (approval number: JMU202412091) on 25th December, 2024. The experiment was conducted between 2nd January, 2025 to 22nd March 2025. All participants provided written informed consent, including consent for data publication.

The research employed three devices: a heart rate monitor, a health monitor, and an electroencephalograph (EEG). The present analysis focuses on heart rate and heart rate variability from the heart rate monitor, and respiration rate, peripheral oxygen saturation (SpO₂), and end-tidal carbon dioxide (EtCO₂) from the health monitor. Results related to brain electrical connectivity, microstates, and power recorded by EEG will be reported separately.

### 2.1 Participants

Sample size was estimated using G*Power (v3.1.9.7) based on a repeated-measures ANOVA with two groups and two measurements. Because directly comparable studies were limited, a conventional medium effect size (*f* = 0.25) was assumed. The analysis was performed with *α* = 0.05, power = 0.80, and a correlation of 0.7 between repeated measures based on our prior research (Liang et al., 2023). The required sample size was 22 participants; anticipating a 20% dropout rate, 26 participants were recruited.

**Figure 1:**
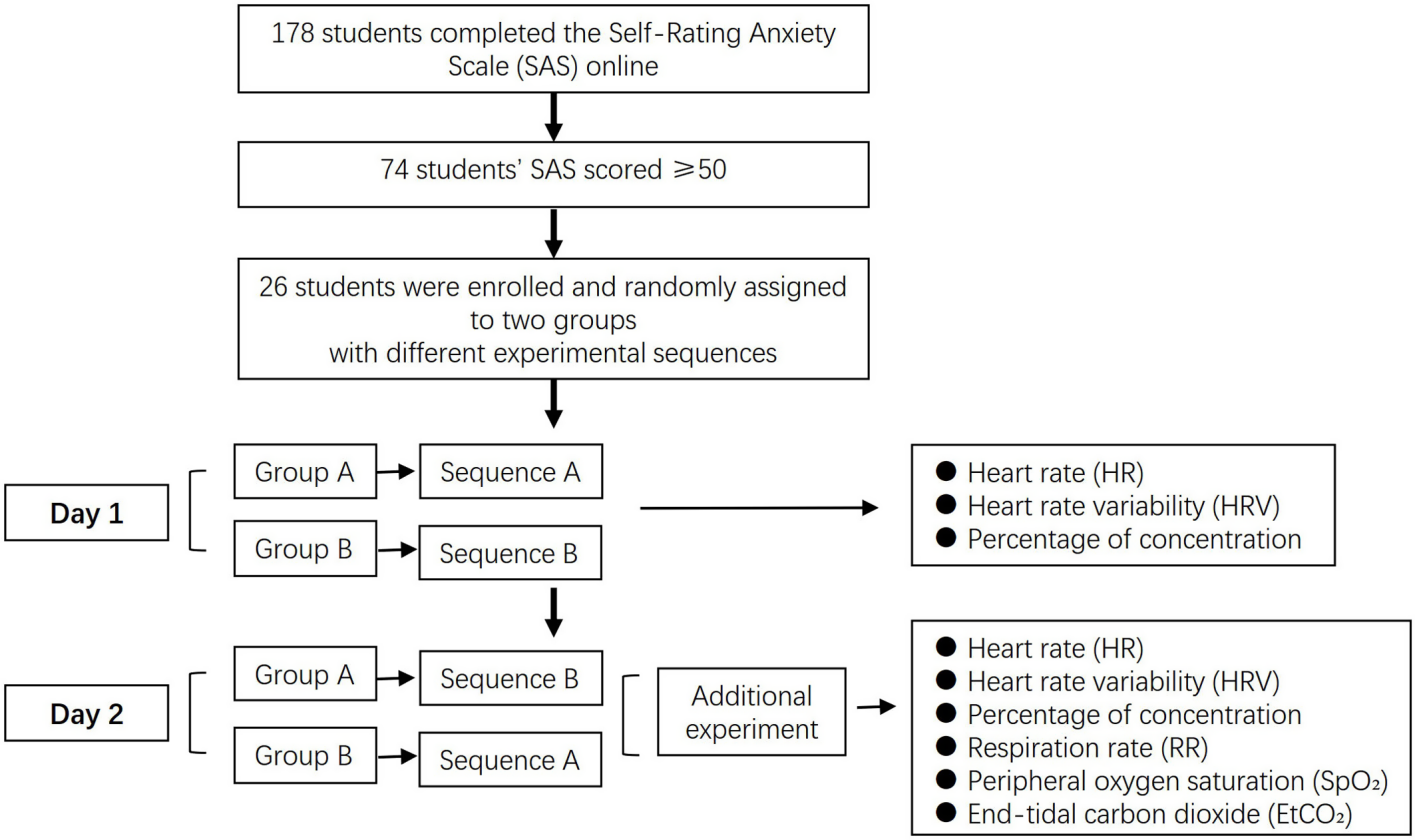
Flowchart of the experimental procedure.

Participants were recruited at Jimei University via poster advertisements and colleague referrals. A total of 178 students completed the Self-Rating Anxiety Scale (SAS) online, of whom 74 scored ≥50, indicated the presence of anxiety (Luo et al., 2022) (Figure 1). These 74 students were contacted sequentially based on the time at which they completed the SAS. Inclusion criteria were: SAS score ≥50 but not subjectively intolerable; BMI between 17.8 and 27.9; self-reported absence of physical illnesses such as hypertension, coronary heart disease, or hyperthyroidism; and no self-reported mental health disorders other than anxiety symptoms. Exclusion criteria included voluntary withdrawal, failure to complete the entire trial, or anxiety symptoms becoming intolerable. Recruitment ceased once 26 participants were enrolled. Only male participants were included to reduce potential physiological variability associated with sex-related differences in autonomic and respiratory responses in this preliminary study.

### 2.2 Learning and evaluation of breathing techniques

Participants were randomly assigned to two groups using SPSS. Uniformly distributed random numbers between 0 and 1 were generated for all participants, who were then reordered according to the generated random numbers and sequentially assigned to two groups with equal sample sizes. Participants were not informed of their group assignment until the experimental session began. They attended a 45-min training session on breathing techniques. A WeChat group was established for sharing instructional videos and Q&A. Participants contacted the instructor to schedule an assessment once they could comfortably perform the two breathing methods. Three assessors evaluated the participants’ performance of the breathing techniques. For mindful breathing, participants were required to focus their attention on sensing their breath at the nose, without deliberately changing its depth or frequency and without judging its quality. For Liuzijue breathing, participants were required to perform the breathing movements correctly in a relaxed manner, with relatively prolonged exhalation accompanied by a low-pitched vocalization of “chui.” They were also required to sequentially guide their attention from the throat to the chest and then to the lower abdomen during vocalization. Three procedural details should be noted. First, participants kept their eyes closed during both Liuzijue breathing and mindful breathing. Second, after each Liuzijue breathing cycle, participants performed two cycles of natural breathing (breathing without intentional control), which is commonly recommended for beginners to reduce the risk of dizziness. Third, participants were instructed to perform the breathing practices in a relaxed and comfortable manner, and no fixed respiratory rate, tidal volume, or breathing depth was imposed.

If at least two assessors agreed the technique met criteria, the formal experiment commenced. Otherwise, participants continued practice until standards were met.

### 2.3 Experimental protocol

The testing took place in a meeting room where the temperature was maintained at approximately 26 °C and the humidity ranged from 40 to 70%. An air conditioner and a temperature-humidity meter (No. 9012, Deli Company, China) were used to monitor and maintain the environmental conditions.

**Figure 2:**
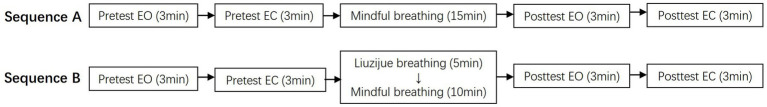
Testing procedure for heart rate and heart rate variability. EO, eyes-open; EC, eyes-closed.

According to group assignments, the experimental sequence for each participant followed a counterbalanced crossover design, with a one-day washout interval between the two experimental sessions. During the testing, participants were requested to quietly sit on the chair with hands resting on the legs. For participants assigned to Sequence A followed by Sequence B, the first day involved 15 min mindful breathing, and the second day included 5 min Liuzijue breathing and 10 min mindful breathing (Figure 2). For participants assigned to Sequence B followed by Sequence A, the first day involved 5 min Liuzijue breathing and 10 min mindful breathing, followed by 15 min mindful breathing on the second day. During the testing, the researchers waited outside the meeting room. The experiments were conducted at the same time on two consecutive days; for example, if the first day started around 10:00 a.m., the second day also began around 10:00 a.m., in order to minimize circadian effect.

After each testing session, participants rated their attentional focus during mindfulness practice.

**Figure 3:**
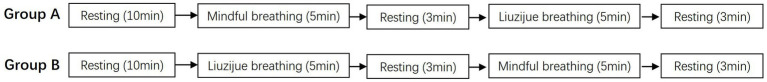
Testing procedure for respiration rate (RR), peripheral oxygen saturation (SpO₂), and end-tidal carbon dioxide.

Following the testing on the second day, participants rested for 10 min, after which a health monitor (Contect-CMS 8000, Qingguangdao, China) was used to measure their end-tidal carbon dioxide (ETCO₂), blood oxygen saturation (SpO₂), and respiratory rate (Figure 3). For the participants in Group A, the test included 5 min of mindful breathing, 3 min of resting with eyes open, 5 min of Liuzijue breathing, and 3 min resting with eyes open. For the participants in Group B, the test included 5 min Liuzijue breathing, 3 min resting with eyes open, 5 min mindful breathing, and 3 min of resting with eyes open. The aim was to assess effects of breathing techniques on physiological parameters and recovery time.

### 2.4 Data collection and processing

#### 2.4.1 Heart rate and heart rate variability data collection and processing

First, the electrodes on the chest strap of the heart rate monitor (Polar H9, Kempele, Finland) were moistened with water. Then it was fastened around the participant’s chest with the sensor placed on the xiphoid process of the sternum. The sensor was connected via Bluetooth to the Elite app (Elite HRV, Inc., Asheville, NC, USA) on an Apple iPhone 15. The beats-to-beats intervals recorded by the heart rate monitor were analyzed using Kubios software (Kubios HRV Standard 3.5.0). We set the beat correction as a low threshold. The rate of beat correction should not be more than 5%, otherwise the participant’s HRV data will not be used (Ballinger, 2004). Data during breathing was reduced to 5 min epochs, to better observe the changes.

#### 2.4.2 Participants’ attentional focus ratings during mindful breathing

After each experimental session, we asked participants: “Please recall your level of attention while practicing mindful breathing and estimate approximately what percentage of focus you maintained. I am referring only to the period when you were performing the mindful breathing.” We recorded the percentages of their reported concentration.

#### 2.4.3 RR, SpO_2_, and EtCO_2_ data collection and processing

SpO_**2**_ was measured on the left index finger with a range of 0–100, 1% resolution, and 1-s update interval. Expired air was collected via a disposable nasal cannula to measure EtCO_**2**_ with a gas flow rate of 50 ± 10 mL/min, 0.1 mmHg resolution (0–69 mmHg), 1-s update interval, and 2–3 s delay. Atmospheric pressure was set to 760 mmHg by default. Calibration was unnecessary, but a 1–2 min warm-up was performed before each participant. Respiration rate was automatically calculated by the monitor.

### 2.5 Statistical analysis

Statistical analyses were conducted using SPSS 27 (IBM, Armonk, NY, USA). Univariate normality was assessed using skewness and kurtosis, with skewness ≥ |2| or kurtosis ≥ |7| indicating non-normal distribution (Post et al., 2023). Data with normal distribution are presented as mean ± standard deviation, whereas skewed data are presented as median (interquartile range).

For HR and HRV outcomes, pre- versus post-test comparisons were conducted within each group. Normally distributed data were analyzed using paired *t*-tests, whereas skewed data were assessed via the Wilcoxon signed-rank test. Since these comparisons were not the main aim of the present study, we did not apply *p*-value correction and did not show effect size.

Generalized Estimating Equations (GEE) were applied for the main statistical analysis, as the models are robust to skewed data and missing observations, and can effectively handle correlated repeated measures (Ballinger, 2004). To account for potential day-to-day variations in participants’ physical or mental states, values were normalized to the pre-test eyes-opened baseline using the formula: Normalized Value = Posttest Value/Pretest Value×100 (Ballinger, 2004). The interaction and main effects were tested using four post-intervention time points (6–10 min mindful breathing, 11–15 min mindful breathing, 3 min post-test eyes-opened, and 3 min post-test eyes-closed). An unstructured working correlation matrix was specified, and the model type was set as either normal distribution or normal distribution with log link, according to the data distribution. For multiple comparisons across HR, RMSSD, and HF (three comparisons in total), we applied the Benjamini–Hochberg false discovery rate (FDR) correction to control false positives while avoiding excessive statistical conservatism (Benjamini and Hochberg, 1995). The adjusted *p*-values were calculated using the standard equation: $p\;(k)\mathrm{adj}=p\frac{k}{m}$ , where *p*(*k*) denotes the adjusted *p*-value, *k* is the rank of the raw *p*-value, and m is the total number of tests. The effect size for GEE results was reported as the regression coefficient (*β* value). The simple effects at each time point were examined using paired-sample *t*-tests or Wilcoxon signed-rank tests, depending on the data distribution.

Paired-samples *t*-tests were used to compare participants’ self-rated concentration during mindful breathing between the two sessions, as well as to analyze RR, SpO₂ and EtCO₂ and test simple effects at each time point.

Significance levels were set at *p* < 0.05 and *p* < 0.01.

## 3 Results

### 3.1 Participants’ basic information

Twenty-four participants completed both testing sessions, and their baseline characteristics are shown in Table 1. The students required an average of 13 days (13.0 ± 2.79 days) to become proficient in the two breathing techniques, whereas mindful breathing was learned within 1 day.

**Table 1:** Students’ basic information.

Characteristic	Value
Number (person)	24
Age (year)	20.5 ± 1.72
Body height (cm)	176 ± 5.40
Body weight(kg)	70.5 ± 7.74
BMI(Kg/m^2^)	22.7 ± 2.33
SAS scores	56.7 ± 7.94
Ethnicity presence	Han (Chinese)

### 3.2 Heart rate and heart rate variability results

Two of the 24 participants had missing data due to recording failure, and no data imputation was performed.

#### 3.2.1 Heart rate results

Pre-test HR did not differ between the two sequences (*t* = −0.255, *p* = 0.801), indicating comparable baselines and no apparent carryover effect.

**Figure 4:**
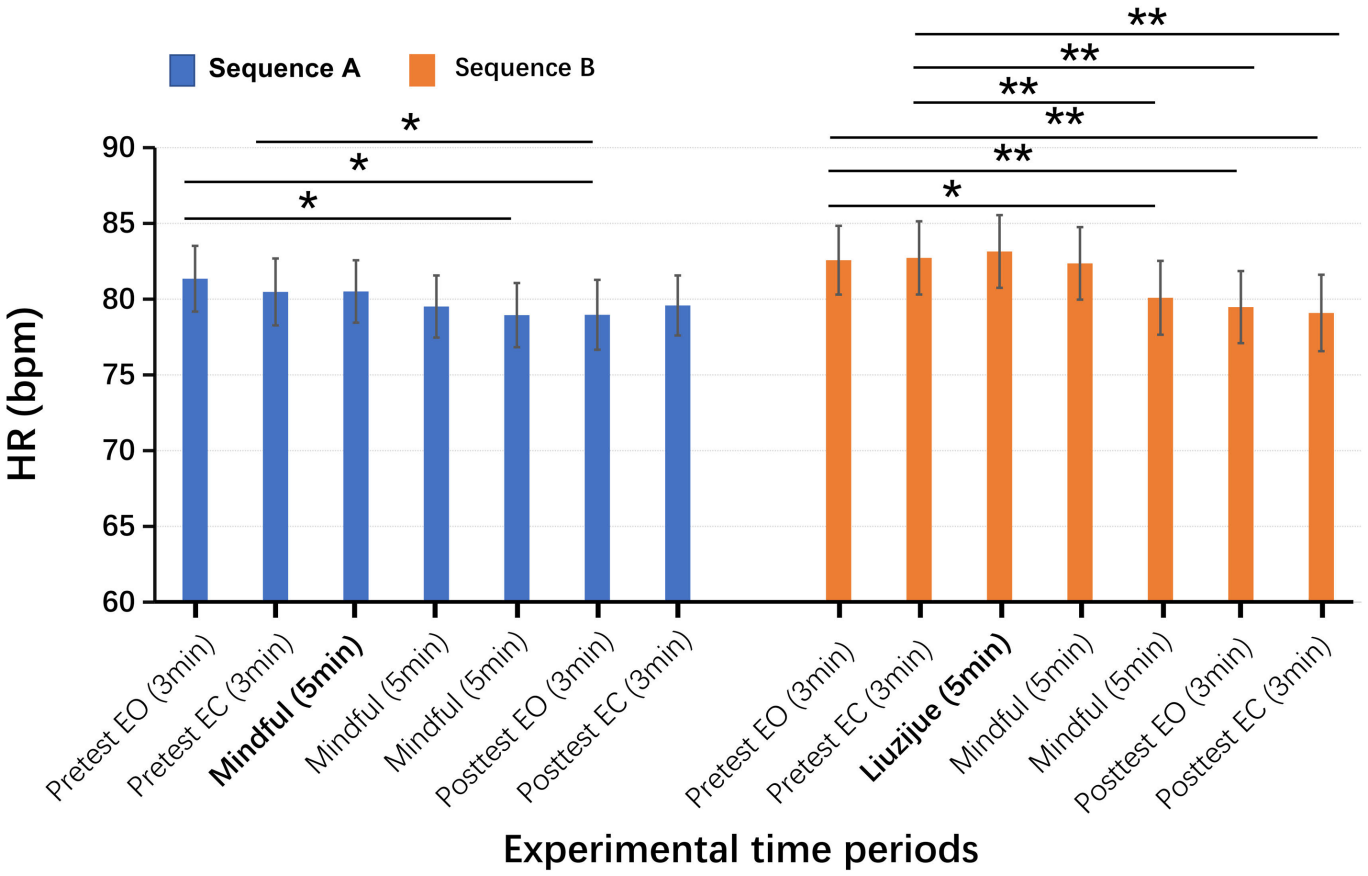
Comparison of heart rate between the pre-test and subsequent periods. * < 0.05, ** < 0.01; EC, eyes-closed; EO, eyes-open; Liuzijue, Liuzijue breathing; Mindful, mindful breathing.

For the within-group comparisons in Sequence A, heart rate during the 10–15 min mindful breathing period and the post-test eyes-open period was significantly lower than during the pre-test eyes-open condition (*t* = 2.554, *p* = 0.018; *t* = 2.570, *p* = 0.017; Figure 4). Compared with the pre-test eyes-closed condition, heart rate during the 10–15 min mindful breathing period was also significantly lower (*t* = 2.274, *p* = 0.033). In Sequence B, heart rate during the 10–15 min mindful breathing period and both post-test periods was significantly lower than during the pre-test eyes-open condition (*t* = 2.358, *p* = 0.027; *t* = 3.465, *p* = 0.002; *t* = 3.342, *p* = 0.003). Similar reductions were observed compared with the pre-test eyes-closed condition (*t* = 3.269, *p* = 0.003; *t* = 4.841, *p* < 0.001; *t* = 4.648, *p* < 0.001).

**Figure 5:**
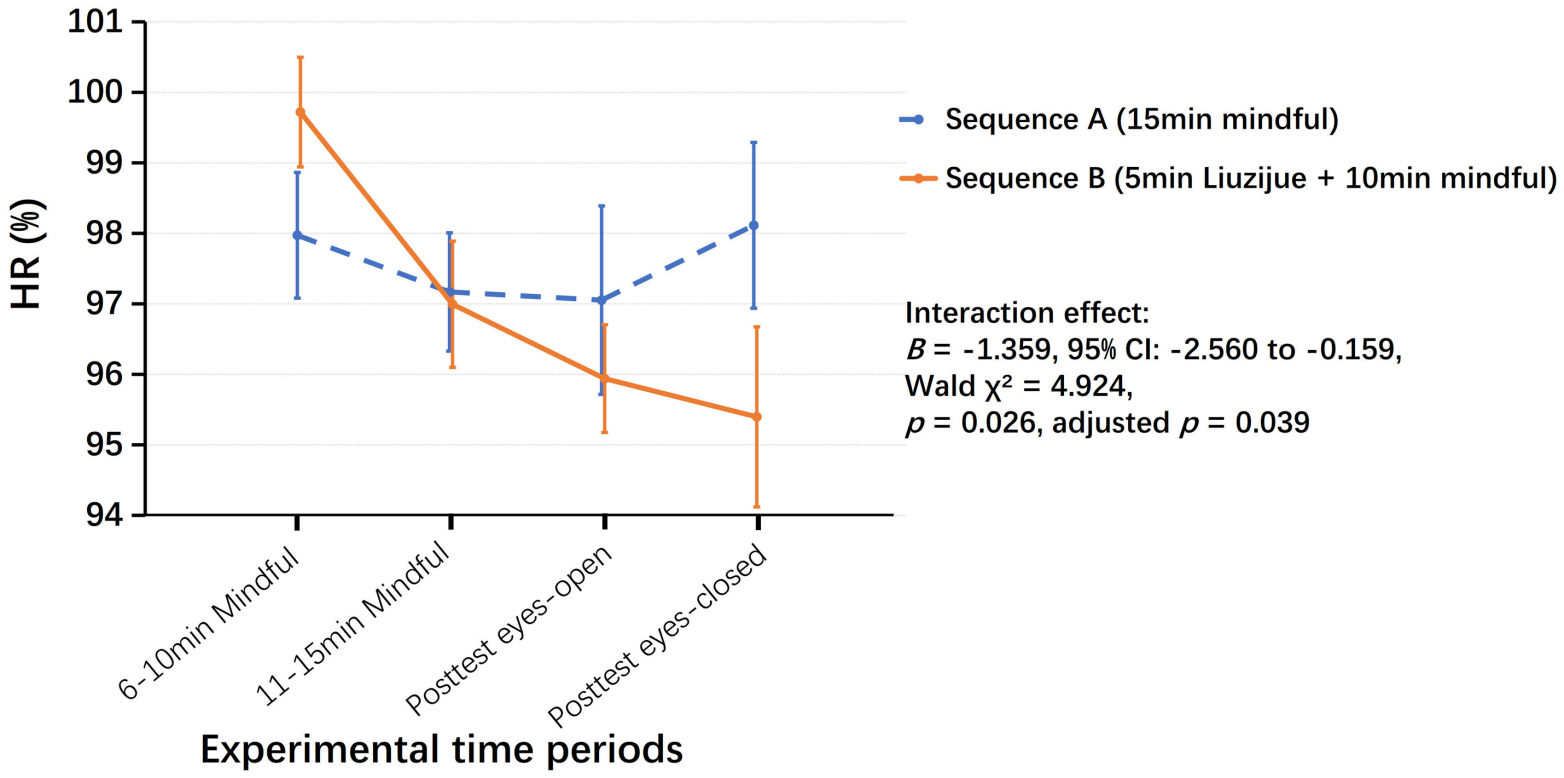
Changes in normalized heart rate in the two sequences. Liuzijue, Liuzijue breathing; Mindful, mindful breathing. Normalized heart rate = relative change from baseline (Post/Pre*100).

For the main statistical analysis, the decrease in heart rate was significantly greater in the Liuzijue plus mindful breathing group than in the mindful breathing group, as indicated by a significant interaction effect (Figure 5). Neither the main effects of time and intervention nor the simple effects reached statistical significance (all *p* > 0.05).

#### 3.2.2 Time-domain (RMSSD) results in heart rate variability

Participants’ HRV, as measured by RMSSD, also did not differ significantly between the two experimental days (*t* = −0.108, *p* = 0.915).

**Figure 6:**
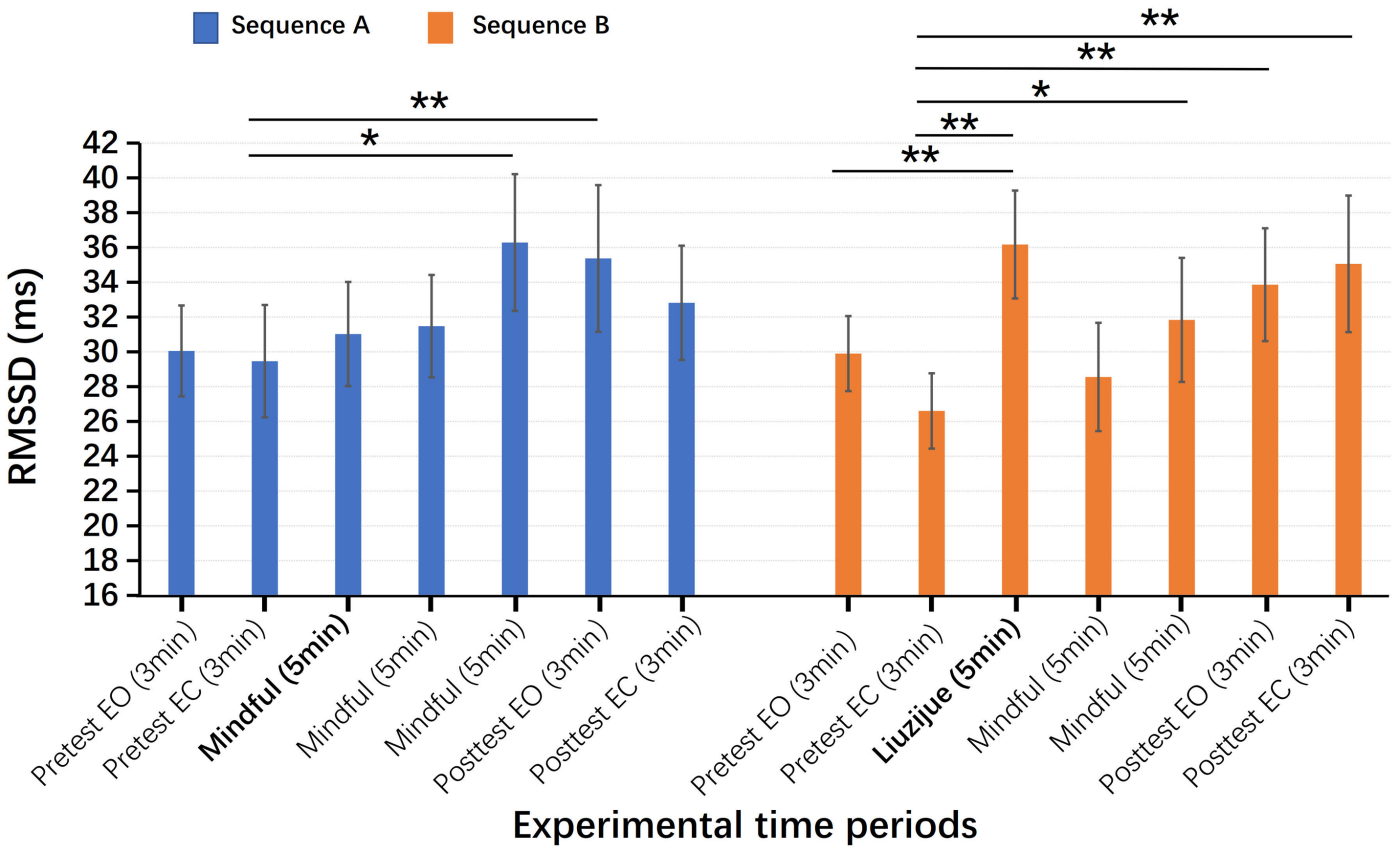
Comparison of HRV (RMSSD) between the pre-test and subsequent periods. * < 0.05, ** < 0.01. EC, eyes-closed; EO, eyes-open; Liuzijue, Liuzijue breathing; Mindful, mindful breathing.

For the within-group comparisons in Sequence A, no significant differences were observed relative to the pre-test eyes-open condition (Figure 6). Compared with the pre-test eyes-closed condition, RMSSD was significantly higher during 10–15 min of mindful breathing and the post-test eyes-open period (*t* = −2.616, *p* = 0.016; *t* = −3.337, *p* = 0.003). In Sequence B, only Liuzijue breathing showed significantly higher RMSSD than the pre-test eyes-open condition (*t* = −2.837, *p* = 0.010). Relative to the pre-test eyes-closed condition, RMSSD was significantly elevated during Liuzijue breathing, 10–15 min of mindful breathing, and both post-test periods (*t* = −4.834, *p* < 0.001; *t* = −2.089, *p* = 0.048; *t* = −3.453, *p* = 0.002; *t* = −2.920, *p* = 0.008).

**Figure 7:**
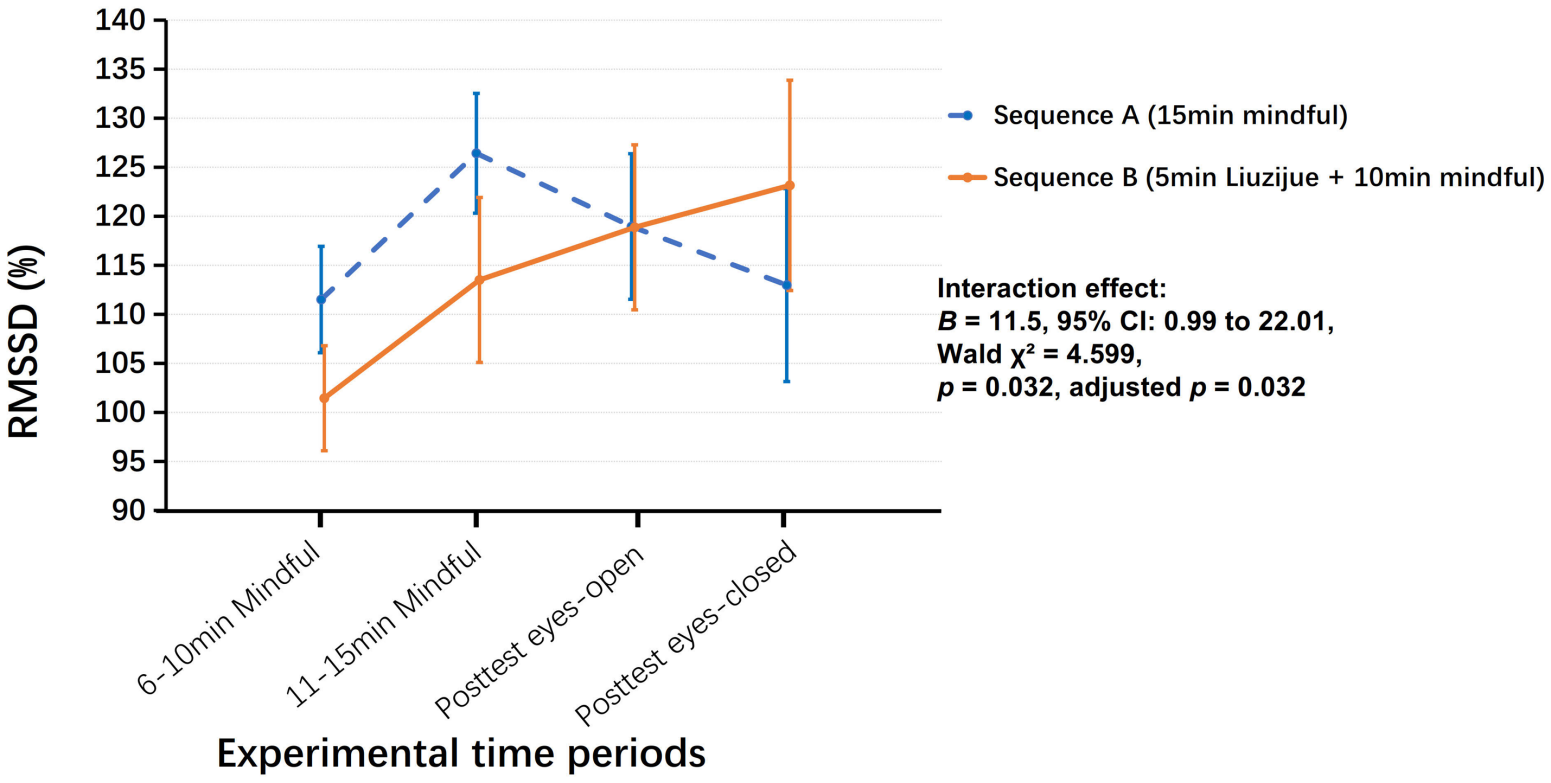
Changes in normalized RMSSD of heart rate variability in the two sequences. Liuzijue, Liuzijue breathing; Mindful, mindful breathing. Normalized RMSSD, relative change from baseline (Post/Pre*100).

As shown in Figure 7, the increase in RMSSD was significantly greater in the Liuzijue plus mindful breathing group than in the mindful breathing group, as indicated by a significant interaction effect. Neither the main effects of time and intervention nor the simple effects reached statistical significance (all *p* > 0.05).

#### 3.2.3 Frequency-domain (HF) results in heart rate variability

**Figure 8:**
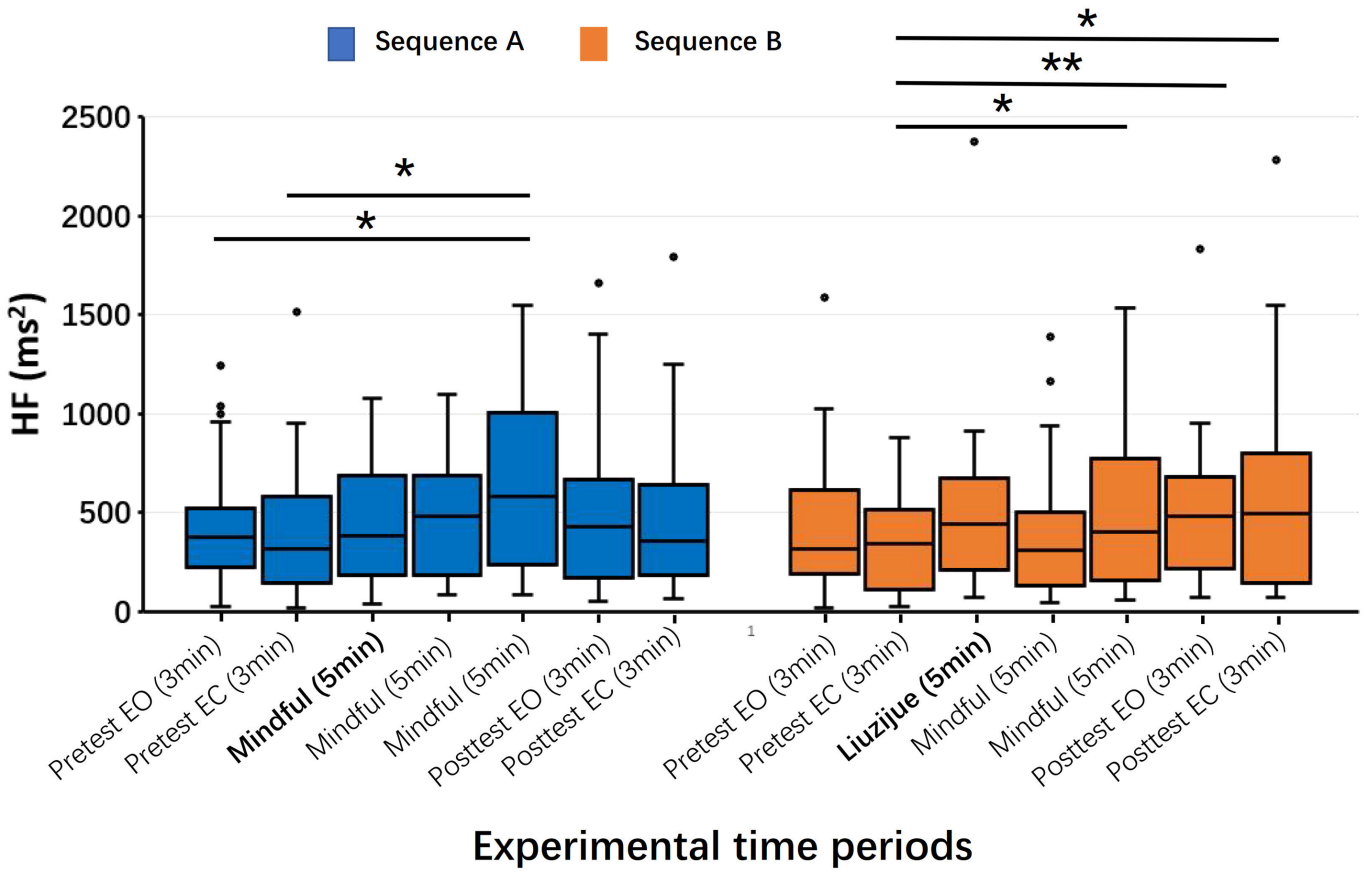
Comparison of HRV (HF) between the pre-test and subsequent periods. * < 0.05, ** < 0.01. EC, eyes closed; EO, eyes-open; Liuzijue, Liuzijue breathing; Mindful, mindful breathing.

For the within-group comparisons in Sequence A, only the 10–15 min mindful breathing period showed significantly higher HF than the pre-test eyes-open condition (*z* = −2.190, *p* = 0.029). Similarly, compared with the pre-test eyes-closed condition, HF was significantly higher during the 10–15 min mindful breathing period (*z* = −2.464, *p* = 0.014) (Figure 8). In Sequence B, no significant differences were observed relative to the pre-test eyes-open condition. Compared with the pre-test eyes-closed condition, HF was significantly elevated during Liuzijue breathing, 10–15 min of mindful breathing, and both post-test periods (*z* = −3.086, *p* = 0.002; *z* = −2.171, *p* = 0.030; *z* = −2.859, *p* = 0.004; *z* = −2.555, *p* = 0.011).

**Figure 9:**
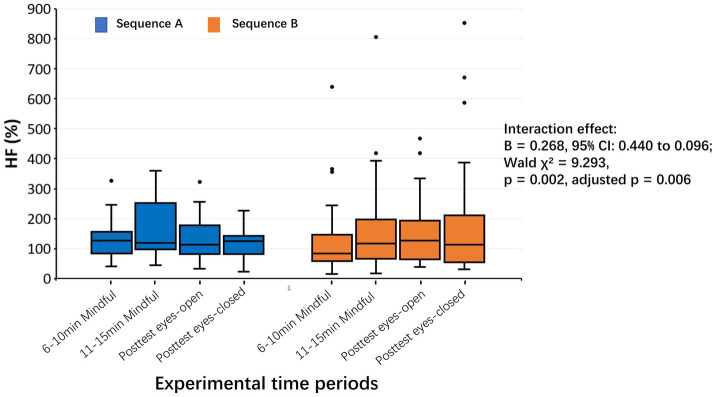
Changes in normalized HF of heart rate variability in the two sequences. Liuzijue, Liuzijue breathing; Mindful, mindful breathing. Normalized HF, relative change from baseline (Post/Pre*100).

Due to the skewed distribution of the data, a normal distribution with a log link function was applied in the GEE analysis. A significant interaction effect was observed (Figure 9), indicating a positive difference between HRV (HF) change rates of the Liuzijue plus mindful breathing group and mindful breathing only group. A decline of HRV (HF) was observed in the mindful only group, as indicated by the significant time effect (*B* = −0.169, CI = −0.310 to −0.028, Wald *χ*^2^ = 5.516, *p* = 0.019). The subsequent analysis revealed a marginally significant increase in HRV (HF) of the Liuzijue plus mindful breathing group (*B* = 0.099, 95% CI: <0.001 to 0.197; Wald *χ*^2^ = 3.847, *p* = 0.050).

### 3.3 Participants’ concentration during mindful breathing

**Figure 10:**
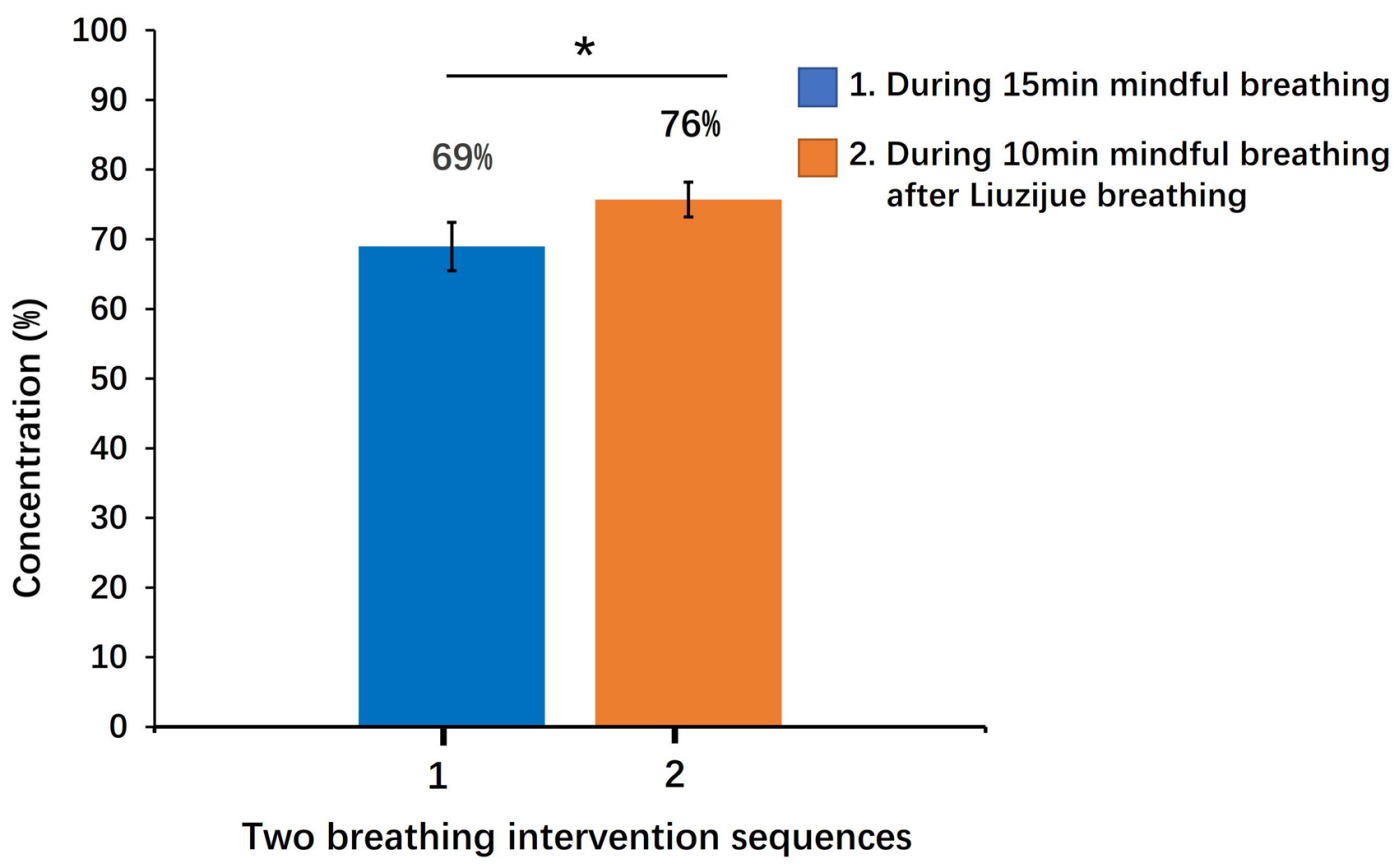
The result of participant’s concentration during mindful breathing.

Among the 24 participants, the concentration percentage during 10 min mindful breathing after Liuzijue breathing was significantly higher than during 15 min mindful breathing (76 ± 3 vs. 69 ± 4; *t* = −2.514, *p* = 0.019) (Figure 10).

### 3.4 Breathing-related physiological parameter results

#### 3.4.1 Respiration rate results

**Figure 11:**
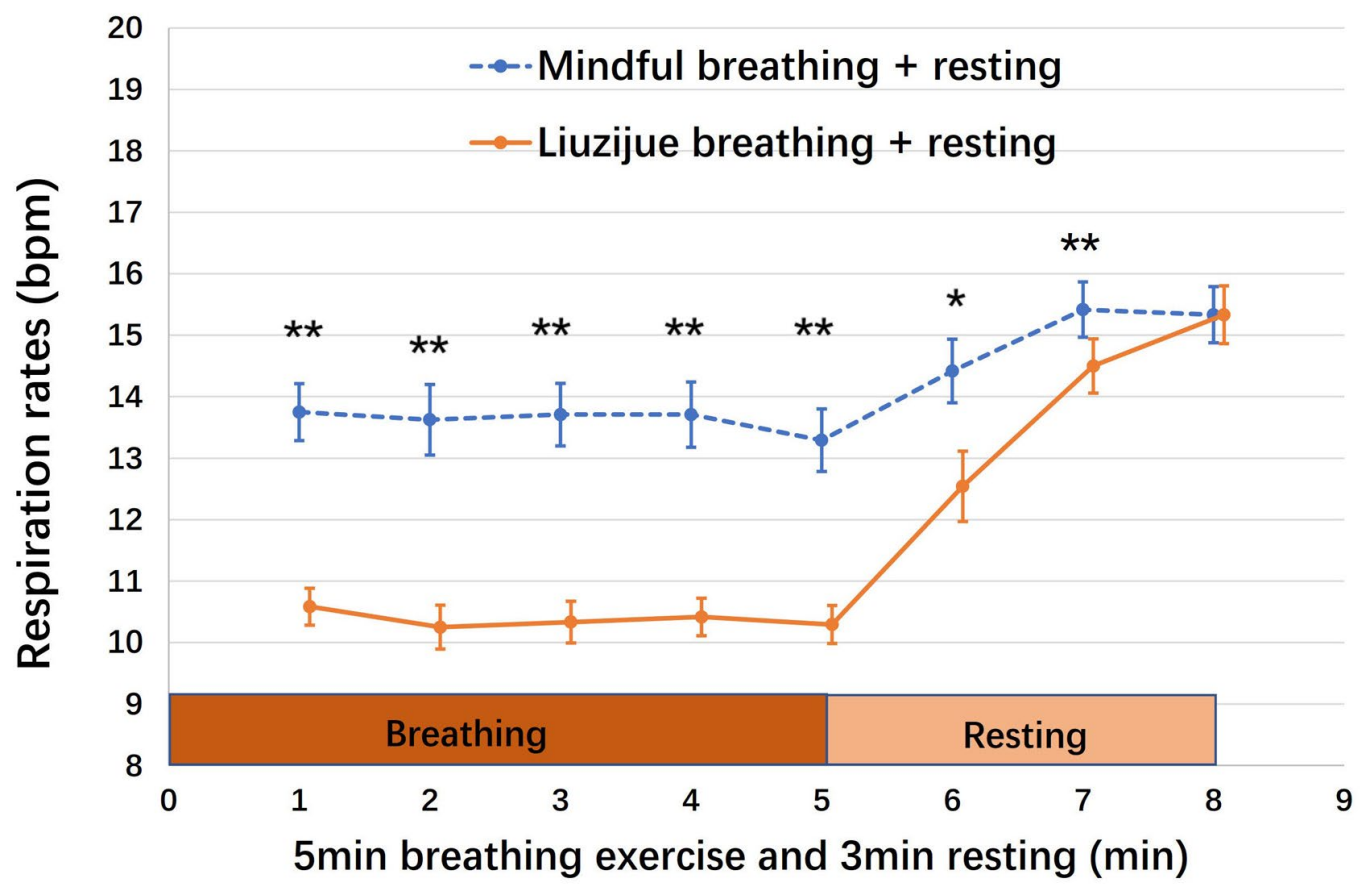
The results of respiration rates (RR). * < 0.05, ** < 0.01.

Figure 11 shows respiration rates (bpm) was higher during mindful breathing (14 ± 3) than Liuzijue breathing (10 ± 2) across the 5 min breathing phase (*t* > 5.789, *p* < 0.001). Significant differences persisted during the first 2 min of rest (minute 1: *t* = 2.532, *p* = 0.019; minute 2: *t* = 2.882, *p* = 0.008) but converged by minute three.

#### 3.4.2 Peripheral oxygen saturation results

**Figure 12:**
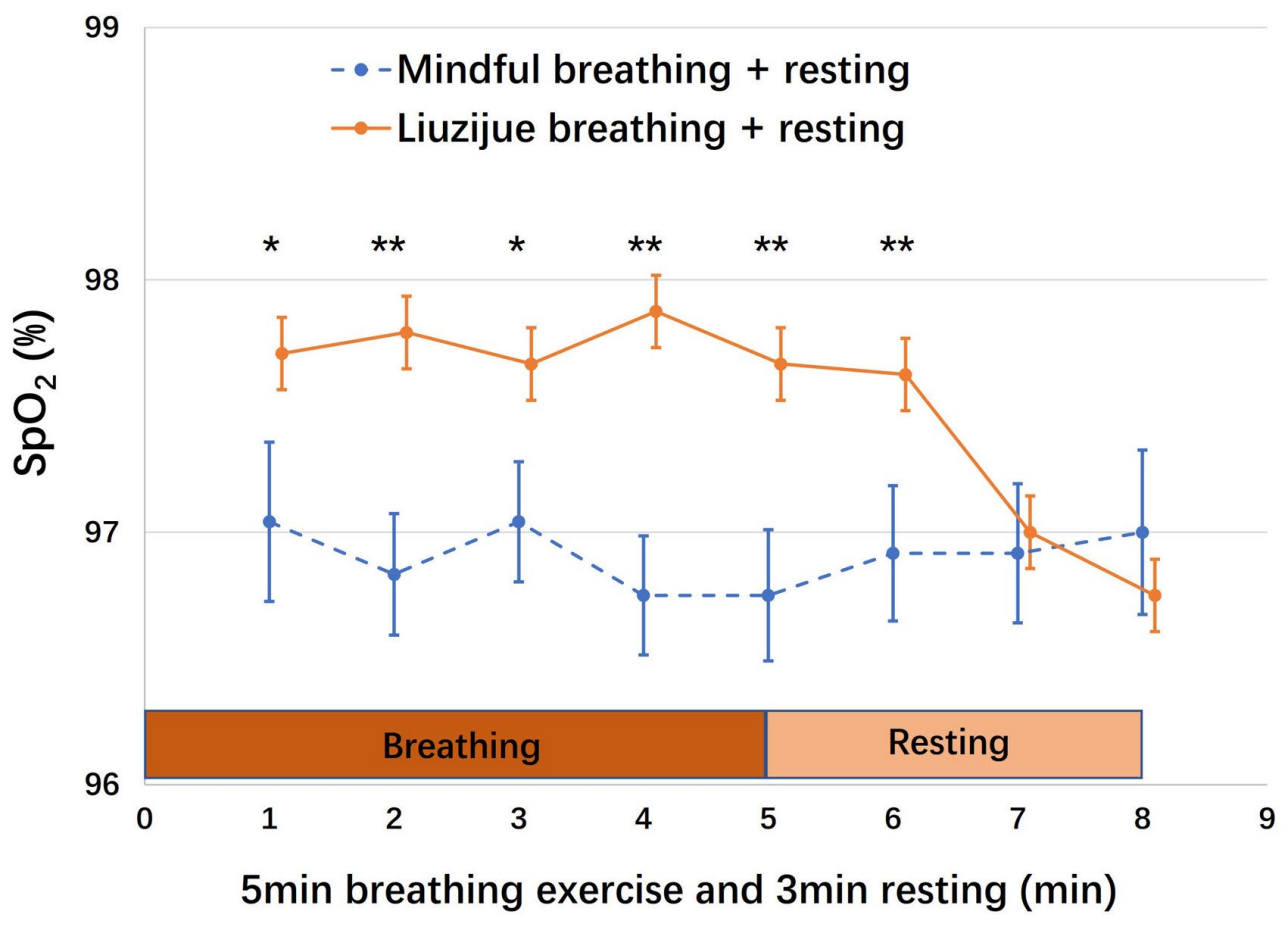
The results of peripheral oxygen saturation (SpO₂).

As shown in Figure 12, peripheral oxygen saturation (SpO₂, %) was consistently greater in the Liuzijue breathing condition during breathing (98 ± 1 vs. 97 ± 2; *t* > 2.713, *p* < 0.012) and remained significantly elevated at the first minute of rest (*t* = −4.303, *p* < 0.001), with differences diminishing thereafter. Although the differences were small, approximately 1%, they were consistent, and it is physiologically influential.

#### 3.4.3 End-tidal carbon dioxide results

**Figure 13:**
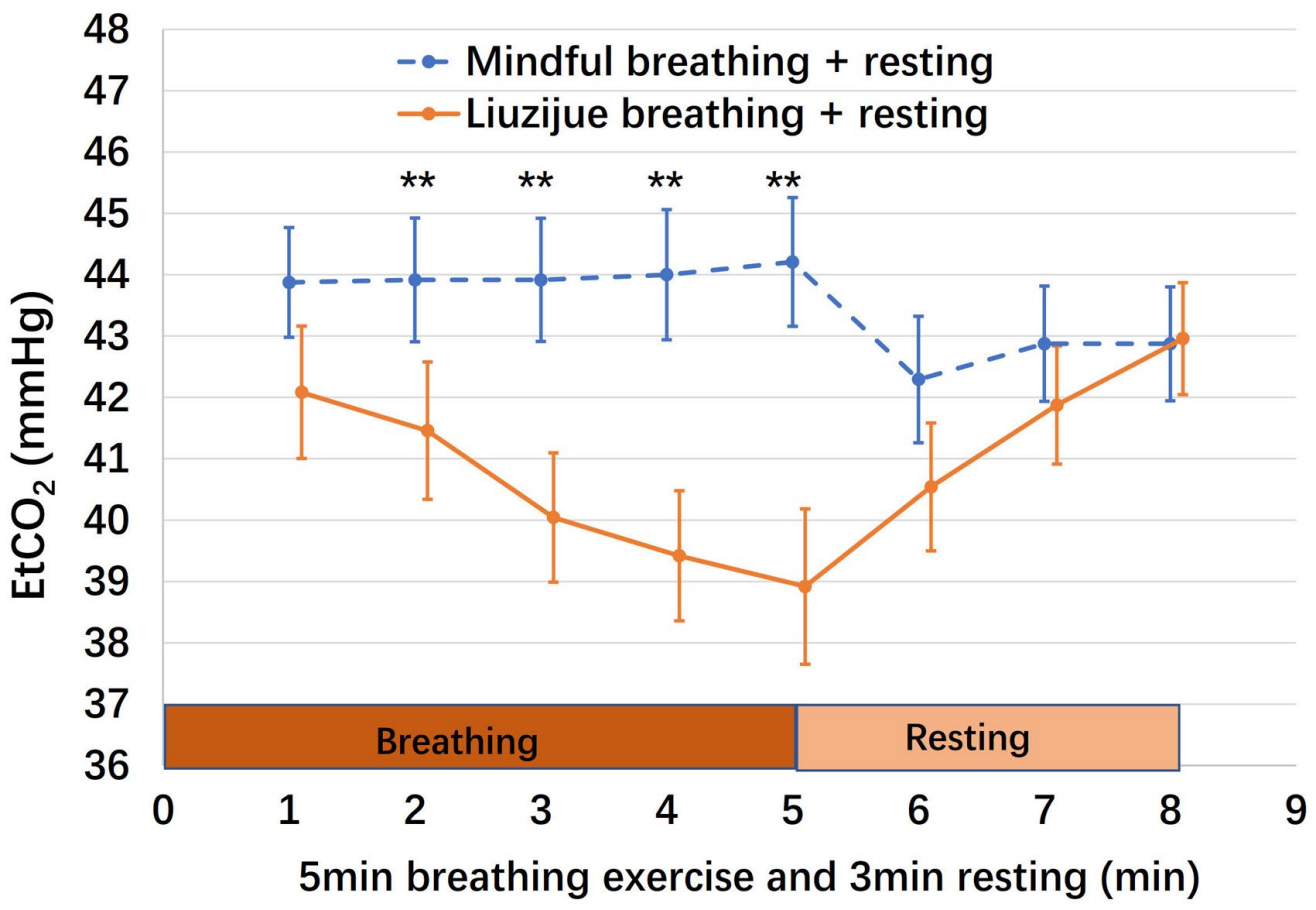
The results of end-tidal carbon dioxide (EtCO_2_).

Figure 13 displays the results of end-tidal carbon dioxide (EtCO₂, mmHg), which was significantly lower during Liuzijue breathing compared to mindful breathing from minute 2 through 5 (*t* > 3.199, *p* < 0.004), decreasing from ~42 to ~39 mmHg, while remaining stable at ~44 mmHg in mindful breathing. During rest, EtCO₂ in the Liuzijue breathing condition rose to levels comparable to mindful breathing within 2 min, with no significant differences throughout the resting period.

## 4 Discussion

This exploratory randomized crossover study revealed that a brief session of Liuzijue breathing followed by mindful breathing produced a more sustained reduction in heart rate (HR) and greater increases in heart rate variability (HRV) compared with mindful breathing alone. These results supported our hypothesis. In addition, the participants’ attentional focus during mindful breathing, along with the RR, SpO₂, and EtCO₂ outcomes observed during both Liuzijue and mindful breathing, may help elucidate the underlying mechanisms of these findings.

The main finding was both interesting and unexpected: following the cessation of mindful breathing, heart rate gradually increased while heart rate variability progressively decreased, indicating a reduction in the parasympathetic activation induced by the practice. In contrast, after the combined Liuzijue and mindful breathing intervention, heart rate continued to decrease and heart rate variability continued to increase, suggesting a more sustained relaxation response. This result slightly deviated from our initial hypothesis, which predicted that physiological indices would return toward baseline after both interventions, albeit to a lesser extent in the combined condition.

We hypothesize that the beneficial effects of Liuzijue breathing arise from its integrated physical and mental regulatory mechanisms involving muscular relaxation, specific respiratory movements, and mindful vocal breathing regulation. During Liuzijue breathing, the lower abdomen contracts inward, the rib cage expands, and the diaphragm descends, creating a noticeable stretching sensation in the mid-back. As exhalation proceeds, these muscles gradually relax, which may resemble elements of progressive muscle relaxation, a technique that has been shown to enhance both psychological and physiological relaxation (Toussaint et al., 2021; Muhammad Khir et al., 2024). The vocalized exhalation in Liuzijue requires directing awareness from the mouth and throat down to the chest and abdomen, a process that similar to body scan meditation. Body scan meditation has been associated with increased respiratory sinus arrhythmia (natural heart rate variability) and reduced anxiety (Ditto et al., 2006; Dreeben et al., 2013). The combination of vocalization and breathing in Liuzijue may exert effects comparable to or potentially stronger than body scan meditation alone, since 12 min of group chanting the sound “Om” has been proven to reduce cortisol levels and anxiety more evidently than its silent repetition (Perry et al., 2024). Thus, we speculate that Liuzijue breathing may induce a general state of bodily relaxation, and this relaxed state may then facilitate subsequent mindfulness practice, potentially contributing to more sustained autonomic regulation, reflected in lower heart rate and higher heart rate variability. These interpretations remain speculative and should be verified in future studies using direct physiological measures.

Another finding is the improved concentration during mindful breathing. Our research found a gradual decrease in EtCO_2_ during Liuzijue breathing, indicating that the ventilation volume was relatively large. Meanwhile, the breathing rate is low—significantly lower than that observed in mindfulness breathing—which suggests that each breath has a larger tidal volume, thereby enhancing ventilation efficiency (due to reduced anatomical dead space and increased alveolar ventilation). Enhanced ventilation leads to higher blood oxygen saturation (Taylor et al., 2025). This increase in blood oxygen saturation may enhances brain function, including cognitive abilities and attentional focus (Post et al., 2023). Improved attentional focus, in turn, facilitates attainment of a mindful state during mindful breathing.

Regarding the safety-related considerations, Liuzijue breathing gradually increased ventilation volume, which implied a rise in pH levels. Within the initial 5 min, pH remained within the normal physiological range, and no participants reported dizziness. However, extending the breathing duration beyond this period may lead to symptoms of hyperventilation. Therefore, it is important to note the following safety considerations: (1) each Liuzijue breath should be followed by two natural breaths, and (2) the breathing duration should be limited to 5 min unless safety is thoroughly validated before increasing the intervention time.

Limitations of this study include the following: First, we did not assess the level of relaxation experienced by participants after completing the entire experiment. Second, this was an acute intervention study. Hence, the long-term impacts of Liuzijue breathing integrated with mindful breathing still warrant further investigation. Third, only male university students were included, which limits the generalizability of the findings to female participants and broader populations. Future studies should include female students to further evaluate the applicability of the findings across sexes. Fourth, further investigations with larger sample size are warranted to confirm our results. Fifth, validated post-intervention psychological assessments were not included, which limits the clinical interpretation of relaxation beyond physiological indicators. Future studies may employ anxiety scales such as the STAI or GAD-7 to better evaluate psychological changes following the intervention. Sixth, attentional focus was assessed using a subjective self-report rating rather than a validated attentional measurement tool. In addition, the experiment was conducted under relatively controlled environmental conditions, including standardized temperature and humidity, which may limit the generalizability of the findings to more pragmatic or real-world settings.

## 5 Conclusion

Brief Liuzijue breathing prior to mindful breathing enhanced the sustainability of relaxation responses in male university students with anxiety symptoms. The improved concentration, reduced RR, and elevated SpO₂ associated with Liuzijue breathing may partly explain this effect.

## Data Availability

The raw data supporting the conclusions of this article will be made available by the authors, without undue reservation.
